# Use of Peroxyacetic Acid as Green Chemical on Yield and Sensorial Quality in Watercress (*Nasturtium officinale* R. Br.) Under Soilless Culture

**DOI:** 10.3390/ijms12129463

**Published:** 2011-12-19

**Authors:** Gilda Carrasco, Claudia Moggia, Ingrid Jennifer Osses, Juan Eugenio Álvaro, Miguel Urrestarazu

**Affiliations:** 1 Facultad de Ciencias Agrarias. Avda. Dos Norte 685, Universidad de Talca, Talca Casilla 747, Chile; E-Mails: gcarrasc@utalca.cl (G.C.); cmoggia@utalca.cl (C.M.); ingrid_osses@live.cl (I.J.O.); 2 Escuela Superior de Ingeniería, Universidad de Almería, Almería 04120, Spain; E-Mail: jam485@ual.es

**Keywords:** watercress, green chemistry, recirculating system, root oxygenation, peroxyacetic mixture, radical oxygenation

## Abstract

The goal of this research was to evaluate the effect of different doses of peroxyacetic acid on the productivity of watercress (*Nasturtium officinale* R. Br.) cultivated hydroponically using a constant nutritive solution. Green chemistry in protected horticulture seeks compatibility with the environment through the creation of biodegradable byproducts. In hydroponics, appropriate doses of peroxyacetic mixtures deliver these byproducts while also oxygenating the roots. Watercress producers who recirculate the nutritive solution can use these mixtures in order to increase oxygenation in the hydroponic system. The experiment took place between August and December 2009, beginning with the planting of the watercress seeds and concluding with the completion of the sensory panels. A completely random design was used, including three treatments and four repetitions, with applications of 0, 20 and 40 mg L^−1^ of the peroxyacetic mixture. Measured variables were growth (plant height, leaf length and stem diameter), yield (weight per plant and dry matter) and organoleptic quality (color and sensory panel). The application of 40 mg L^−1^ of the peroxyacetic mixture had a greater effect on the growth and development of the plants, which reached an average height of 29.3 cm, stem diameter of 3.3 mm and leaf length of 7.6 cm, whereas the control group reached an average height of only 20.2 cm, stem diameter of 1.9 mm and leaf length of 5.7 cm. The application of the peroxyacetic mixtures resulted in an improvement in growth parameters as well as in yield. Individual weights achieved using the 40 mg L^−1^ dose were 1.3 g plant^−1^ in the control group and 3.4 g plant^−1^ in the experimental group (62% yield increase). Sensory analysis revealed no differences in organoleptic quality.

## 1. Introduction

Watercress (*Nasturtium officinale* R. Br.) is a perennial aquatic plant native to Europe that belongs to the Brassicaceae family [1]. Soilless cultivation is often employed in the production of this vegetable, because it is considered a useful technique for the development of leafy vegetables when early maturity is sought [2]. Moreover, this plant is very light sensitive, though it can acclimate to low-light regions by augmenting its physiology and morphology in order to increase photosynthetic efficiency [3]. The production of watercress in Chile is concentrated primarily in the Santiago Metropolitan Area, including 68.5 ha in greenhouses [4]. It is a good source of essential vitamins and minerals and beneficial phytochemicals, such as lutein and zeaxanthin [5].

Green chemistry aims to reduce the flow of chemicals into the environment. To this end, the chemical industry must see to it that chemical processes are not toxic and do not create byproducts that contain contaminants; moreover, the industry should use biodegradable raw materials [6]. The incorporation of green chemistry strategies in protected horticultural practices and in the food industry could lead to the release of biodegradable substances that do not contaminate the environment. In hydroponics, the use of appropriate doses of peroxyacetic mixtures achieves disinfection and improves root oxygenation, which has a positive effect on yield, as well as improving the quality of post-harvest horticultural products [7].

Root aeration is an important factor in obtaining a larger number of plants. Among the methods used to oxygenate the nutritive solution are the use of compressed air and the recirculation of the solution, both of which require pumps to bubble air into the solution. An elevated level of oxygenation favors rapid root growth, which will result in higher yields [8].

Some experiments have obtained good results using various sources of peroxides in soilless cultures [8]. On the other hand, other experiments have reported phytotoxicity at concentrations of 80 mg L^−1^ [9]. This is the case with equilibrium mixtures containing peracetic acid, acetic acid, hydrogen peroxide and water in variable proportions, according to predetermined quantities of acetic acid and hydrogen peroxide [10,11], where the chemical compositions are detailed in Table 1. The peroxyacetic acid (PAA, C_2_H_4_O_3_) forms a mixture of hydrogen peroxide (H_2_O_2_) and acetic acid (CH_3_COOH) dissolved in an aqueous solution, which acts as an oxidizing agent that is more effective than chlorine or chlorine dioxide. However, one of the principal applications of peroxyacetic acid is as a disinfectant used in the food processing industry to control odors and bioparticulates on tool and structural surfaces; for this reason, moreover, it is used as surface protection on post-harvest fresh fruit and vegetables [9]. Peroxyacetic acid is an effective bactericide, sporicide, fungicide and virucide, because it acts across cellular membranes, oxidizing molecules and destroying essential enzymatic systems [12]. For this reason, it is used in agricultural applications, because its breakdown yields no nocive secondary compounds: green chemistry. Disinfection using hypochlorites yields byproducts such as chlorine vapor and chloroform, among others, which have proven to be carcinogenic [7].

Therefore, given that the application of peroxyacetic mixtures enables increased aeration of the nutritive solution and, consequently, increased production, it is necessary to determine the specific dose that will result in higher yield without phytotoxicity. The goal of this project was to determine the effect on watercress of the application of 0, 20 and 40 mg L^−1^ of peroxyacetic acid.

## 2. Material and Method

The research was carried out in the greenhouse on the campus of Talca University in Chile’s Maule Region (35°25′59″ South Latitude, 71°40′00″ West Longitude; altitude: 102 masl) between August 20 and December 14, 2009.

The vegetative material used was watercress plants (*Nasturtium officinale* R. Br.) var. Cresson de Fontaine. The watercress seeds from which the seedlings were obtained were sowed on a mixed substrate: pine compost (organic substrate) and perlite A6 (inorganic substrate), combined at a 3:1 ratio. A solid, crystalline complete fertilizer (18-6-18, N-P-K), sold under the trade name Ultrasol Desarollo [13], was used. In addition, we used other essential elements: magnesium, sulfur, iron, zinc, manganese, molybdenum, boron and copper. Tradecorp AZ and Tradecorp Fe were also applied.

The peroxyacetic mixture described in Table 1 was used. The culture was established on a substrate board with a mixture of composted pine and perlite at a 3:1 ratio. The board measured 1 m × 2 m. The seeds were sowed on moistened substrate. Spacing was 10 cm between rows and 1 cm within rows. The time from sowing to transplant was 40 days; the average height at transplant was 3 cm. In the nutrient solution two commercial doses of a peroxyacetic acid mix (20 and 40 mg L^−1^, equivalent to 2 and 4 mg L^−1^ of pure PAA respectively) were evaluated (Table 1), along with a control treatment. Applications took place in the beginning of experiment and once a week when all the hydroponic nutritive solution was changed.

Twelve baths were set up with the following dimensions: 90 cm long, 90 cm wide and 10 cm deep. Next, a 4-cm layer of nutritive solution was added. The pH, CE and dissolved oxygen were measured weekly.

An entirely random design with four blocks per treatment was used [14]. Vegetative growth and yield parameters were measured and quality variables evaluated by a sensory panel. Each plot (experimental unit) had 280 plants (140 plants for growth and the sensory parameters, respectively).

Treatment effects were evaluated on the basis of three harvests from 36, 49 and 67 days after transplant, respectively. Growth parameters were evaluated at 60 days since transplant. Harvest standard was an average plant height of 15 cm from the base. On the day of the last measurement of each harvest, a sensory panel evaluated general appearance, color, texture and taste of each treatment. In all, there were three sensory panels corresponding to the three harvests of the trial: each panel comprised 40 panelists.

A sensory analysis (Table 2) was carried out with the samples contained in perforated, low-density polyethylene bags corresponding to the three treatments. Sensory panels took place on the ninth day following each harvest in order to evaluate general appearance, color, texture and taste for each treatment. The sensory panels used vegetative material that had been held in a system of perforated, low-density polyethylene bags for eight days.

The growth, yield and sensorial quality variables were subjected to variance analysis using the Statgraphics Centurion statistical program. In those cases where statistical differences existed, Tukey’s HSD test (*P* ≤ 0.05) was used to separate the means.

## 3. Result and Discussion

### 3.1. Vegetative Growth

Plant height, leaf length and stem diameter showed significant differences on the last date of harvest after application of the peroxyacetic acid mixtures, as compared to controls (Table 3). At the same time, there was no difference in the number of leaves during the trials (data not shown).

The variables demonstrated a positive effect in relation to increased oxygenation and, in general. This is also reflected in the measurements taken at harvest. Although no differences were noted at the beginning of the trials, results over time revealed the advantages of using the peroxyacetic acid mixture.

Reference [8] reports that the utilization of oxygenating products augments the availability of oxygen at the plant root, due to the byproducts produced by the reactions of the peroxyacetic mixture. Therefore, it can be concluded that the application of this mixture has a positive effect on morphological development of plants harvested at the end of the trials, since these oxygenation techniques prevent possible aeration problems, increase nutrient absorption efficiency and significantly augment the productivity of the plants [7].

Reference [15] affirms that poor aeration reduces nutrient absorption by as much as 50%. Therefore, the oxygen concentration of the nutritive solution should involve ≥ 3 mg L^−1^ of dissolved oxygen in the nutritive solution, so as not to affect vegetative growth. Oxygenation levels during the trials were above this level (data not shown); levels increased significantly toward the end of the trials.

Higher oxygen levels may have contributed to optimal conditions for plant development. At the same time, as the temperature of the nutritive solution increased, oxygen availability fell, leading to competition between the roots and the microflora for oxygen uptake, the latter appearing to be more successful in obtaining oxygen [15].

### 3.2. Effects of Treatments on Yield Variables

The results obtained during the first two harvests did not show significant differences between treatments. However, there were differences at the final harvest. The 40 mg L^−1^ application resulted in greater weight than the control or the 20 mg L^−1^ dose (Table 4).

The yield variables that were evaluated show that the application of 40 mg L^−1^ of the peroxyacetic mixture resulted in significantly higher values than did treatment at 20 mg L^−1^ or control. The proper addition of peroxyacetic mixtures conditions and renews the irrigation water [16] by improving root oxygenation, which tends to have a positive effect on plant yield [7] (Carrasco and Urrestarazu, 2010). The use of a 40 mg L^−1^ dose of the peroxyacetic mixture achieved a yield improvement of 62% and 67% in fresh and dry weight, respectively, as compared to the control group.

### 3.3. Quality Variables: Sensory Analysis

However, the third panel showed that the panelists distinguished differences in general appearance. The control group exhibited significantly different results than did applications of 20 or 40 mg L^−1^ of the peroxyacetic mixture. A majority judged the control treatment as “very pleasant,” while the 20 and 40 mg L^−1^ applications of the peroxyacetic mixture were evaluated as being “moderately pleasant” (Table 5).

The quality variables analyzed in the three harvests were rated positively overall by the sensory panel. The sensory results coincide with those obtained by [9] for peppers, tomatoes and cucumbers disinfected with peroxyacetic mixtures.

## 4. Conclusion

The application of different peroxyacetic mixtures positively influenced vegetative growth and yield parameters in water cress, including plant height, leaf length and stem diameter. Better results were obtained using a dose of 40 mg L^−1^). Compared to the control, there were increases in plant height, leaf length and stem diameter of 31%, 25% and 31%, respectively. The application of 40 mg L^−1^was the treatment producing the best results for plant weight at a particular harvest, resulting in 62% and 67% increases in fresh and dry weights, respectively. In general, the application of peroxyacetic mixtures did not affect the organoleptic qualities of the watercress.

## Figures and Tables

**Table 1:** Chemical composition of the ingredients in a peroxyacetic mixture.

Component	Formula	Percentage by weight
Peroxyacetic acid	CH_3_OOOH	12
Hydrogen peroxide	H_2_O_2_	18.5
Acetic acid	CH_3_OOH	20
Water	H_2_O	Remainder

Source: Solvay, 2005.

**Table 2:** Ranking of categories utilized to evaluate the organoleptic qualities of watercress grown hydroponically with different doses of a peroxyacetic mixture. The parameters measured by three sensory panels were: general appearance, taste, color and texture.

Ranking	Category
1	Extremely pleasant
2	Very pleasant
3	Somewhat pleasant
4	Slightly pleasant
5	Indifferent
6	Slightly unpleasant
7	Somewhat unpleasant
8	Very unpleasant
9	Extremely unpleasant

**Table 3:** Growth parameters in watercress plants grown hydroponically with different doses of a peroxyacetic mixture (PM, mg L^−1^) at 60 day since transplant.

PM	Height (cm)	Leaf length (cm)	Stem diameter (mm)
0	20.2 a	5.7 a	1.9 a
20	29.1 b	6.0 a	2.5 b
40	29.3 b	7.6 b	3.3 c

Values denoted by letters indicate significant differences at 5%. Tukey’s test (*P* ≤ 0.05). Average values from four replications.

**Table 4:** Yield of three harvests of watercress plants (g plant^−1^) grown hydroponically with different doses of a peroxyacetic mixture (PM, mg L^−1^).

	Harvest		
PM	1	2	3
	
	Fresh weight		
0	5.8	1.5	1.3 a
20	6.2	1.6	1.9 a
40	6.9	2.5	3.4 b
	n.s.	n.s.	*
	**Dry weight**		
0	0.50	0.10 a	0.11 a
20	0.62	0.14 a	0.21 b
40	0.63	0.24 b	0.34 c
	n.s.	*	*

Values denoted by letters indicate significant differences at 5%. Tukey’s HSD test (*P* ≤ 0.05). n.s. denotes not significant. Average values from four replications.

**Table 5:** The effect of different doses of a peroxyacetic mixture (PM, mg L^−1^) on four sensory variables in hydroponic watercress, as measured by three sensory panels from harvests at 36, 49 and 67 days to transplant respectively.

	General Appearance	Taste	Texture	Color
PM	Harvest 1			
0	3.1	3.9	2.9	2.7
20	3.3	3.5	2.7	2.7
40	3.3	4.3	3.2	3.0
	n.s.	n.s.	n.s.	n.s.
	Harvest 2			
	
0	3.2	3.4	3.3	2.9
20	3.2	3.3	3.1	3.0
40	2.8	3.5	2.9	2.6
	n.s.	n.s.	n.s.	n.s.
	Harvest 3			
	
0	2.7 a	3.2	3.4	2.7
20	3.4 b	4.0	3.5	3.4
40	3.3 b	4.0	3.4	3.2
	*	n.s.	n.s.	n.s.

Values denoted by letters indicate significant differences at 5%. Tukey’s test (*P* ≤ 0.05). Average values from four replications. n.s. denotes not significant. Values utilized according to the hedonic scale (Table 2).
